# Data of intracellular insulin protein reduced by autophagy in INS-1E cells

**DOI:** 10.1016/j.dib.2016.07.008

**Published:** 2016-07-14

**Authors:** Han Sung Kim, Yeong-Min Yoo

**Affiliations:** Department of Biomedical Engineering, Yonsei-Fraunhofer Medical Device Laboratory, Yonsei University, Wonju, Gangwon-do 26493, Republic of Korea

**Keywords:** Insulin, Autophagy, 2-Deoxy-D-glucose, Rapamycin, INS-1E Insulinoma cells

## Abstract

Autophagy appears to be involved in maintaining normal intracellular insulin content by accelerating the insulin degradation rate in β-cells (Marsh et al., 2007) [1]. 2-deoxy-d-glucose (2-DG) is metabolized by hexokinase, and acts as an inhibitor of glycolysis. 2-DG triggers glucose deprivation without altering other nutrients or metabolic pathways (Aghaee et al., 2012) [2], and appears to be an ideal tool for studying autophagy. Rapamycin induced upregulation of autophagy in both cultured isolated islets and pancreatic β-cells (Tanemura et al., 2012) [3]. Here, we examined that 2-DG or rapamycin-induced autophagy may decrease the production of intracellular insulin in INS-1E insulinoma cells. Data showed that autophagy was increased by 2-DG or rapamycin by Western blotting and Immunofluorescence staining analyses. Also, intracellular insulin decreased by 2-DG or rapamycin. Furthermore, the autophagy inhibitors, bafilomycin A1 and/or 3-methyladenine, in the presence or absence of 2-DG or rapamycin increased intracellular insulin in INS-1E insulinoma cells.

## Specifications Table

**Table:** 

Subject area	*Biochemistry*
More specific subject area	*Insulin synthesis, autophagy*
Type of data	*Figure*
How data was acquired	*Immunofluorescence staining, Western blotting*
Data format	*Analyzed*
Experimental factors	*Autophagy in INS-1E insulinoma cells were induced by treatment of 2-DG or rapamycin, and administration of bafilomycin A1 or 3-methyladenine inhibited autophagy, resulted in increase of intracellular insulin levels.*
Experimental features	*Autophagy showed Western blotting of LC3 II, Beclin 1, and insulin proteins and Immunofluorescence staining of LC3-positive granules or lysosomal-associated membrane protein 2 (Lamp2) under the conditions of 2-DG or rapamycin, and /or bafilomycin A1 or 3-methyladenine treatments.*
Data source location	*Wonju, Gangwon-do, Republic of Korea*
Data accessibility	*All data are provided with this article*

## Value of the data

• The data provide 2-DG treatment increase autophagy and reduce intracellular insulin synthesis.
• The data show that rapamycin increase autophagy and subsequently result in a decrease in insulin production.
• This Data could give a base for the detection of intracellular insulin in both cultured isolated islets and pancreatic β-cells by Western blotting analysis.
• The data support the development of intracellular insulin detection by drug-induced autophagy.

## 1 Data

**Fig. 1:**
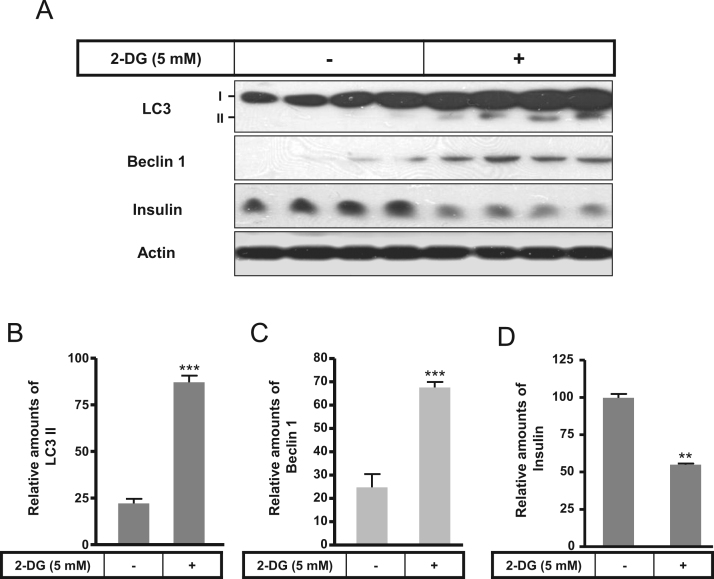
Expression of LC3, Beclin 1, and insulin in the presence or absence of 2-DG in rat INS-1E insulinoma cells. INS-1E cells were incubated in RPMI 1640 medium supplemented with 2% FBS with or without 2-DG (5 mM) for 24 h at 37 °C with 5% CO_2_. LC3, Beclin 1, and insulin were measured by Western blot (A). The relative amounts of LC3 (B), Beclin 1 (C), and insulin (D) were quantified as described in the Methods section. Data represent mean±SD of three experiments. ^***^p<0.001 vs. 2% FBS.

**Fig. 2:**
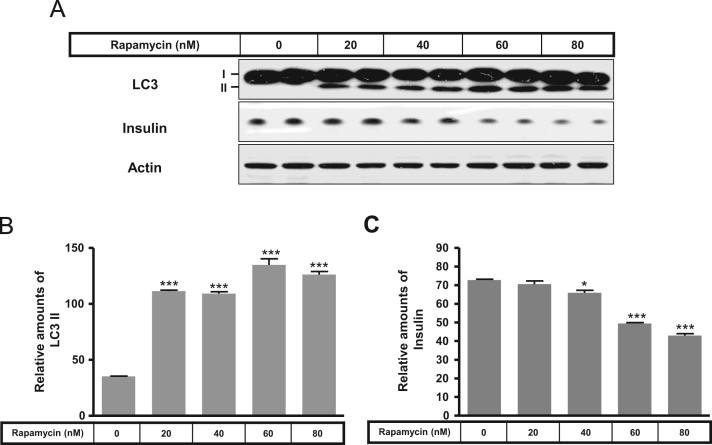
Expression of LC3 and insulin in the presence or absence of rapamycin, in rat INS-1E insulinoma cells. INS-1E cells were incubated in RPMI 1640 medium supplemented with 2% FBS with or without rapamycin (20–80 nM) for 24 h at 37 °C with 5% CO_2_. LC3 and insulin were measured by Western blot (A). The relative amounts of LC3 (B) and insulin (C) were quantified as described in the Methods section. Data represent mean±SD of three experiments. *p<0.05 and ***p<0.001 vs. 2% FBS.

**Fig. 3:**
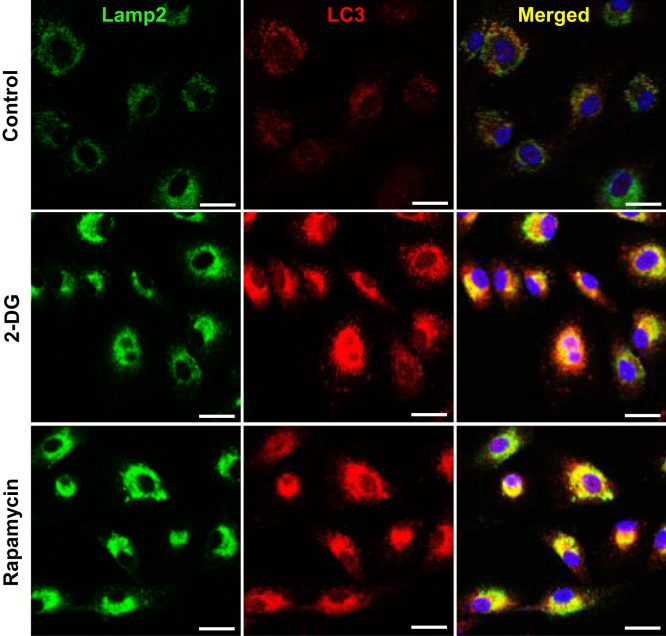
Expression of cellular insulin following bafilomycin A1 or 3-methyladenine treatment with 2-DG in rat INS-1E insulinoma cells. INS-1E cells were incubated in RPMI 1640 medium supplemented with 2% FBS with or without 2-DG (5 mM) for 24 h, and with or without bafilomycin A1 (100 nM) or 3-methyladenine (10 mM) for 6 h at 37 °C with 5% CO_2_. Insulin was measured by Western blot (A) and the relative amounts of insulin (B) were quantified as described in the Methods section. Data represent mean±SD of three experiments. *p<0.05, **p<0.01, and ***p<0.001 vs. 2% FBS; ^###^*p*<0.001, 2-DG vs. 2-DG+bafilomycin; ^&&^*p*<0.001, 2-DG vs. 2-DG+3-methyladenine.

**Fig. 4:**
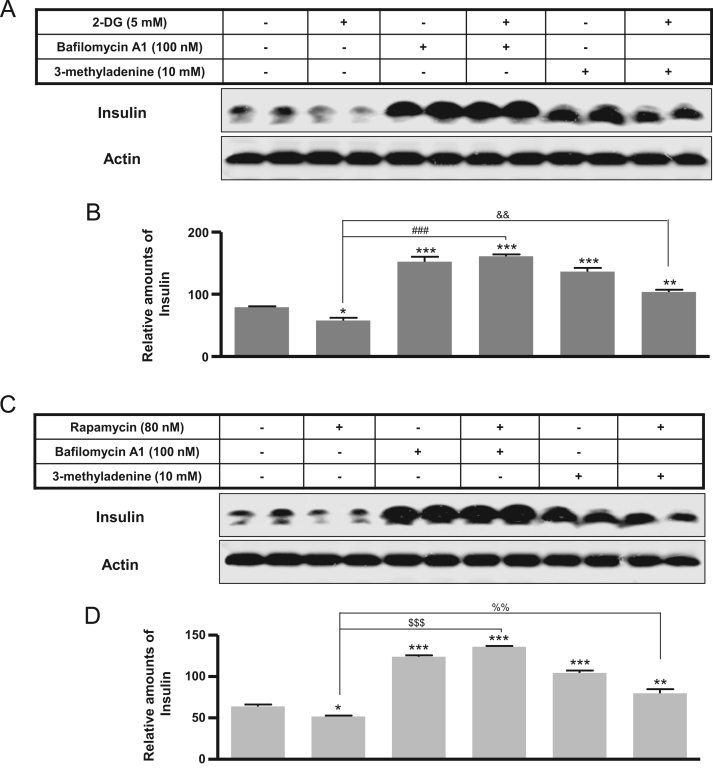
Expression of cellular insulin following bafilomycin A1 or 3-methyladenine treatment with rapamycin in rat INS-1E insulinoma cells. INS-1E cells were incubated in RPMI 1640 medium supplemented with 2% FBS with or without rapamycin (80 nM) for 24 h, and with or without bafilomycin A1 (100 nM) or 3-methyladenine (10 mM) for 6 h at 37 °C with 5% CO_2_. Insulin was measured by Western blot (A) and the relative amounts of insulin (B) were quantified as described in the Methods section. Data represent mean ± SD of three experiments. *p<0.05, **p<0.01, and ***p<0.001 vs. 2% FBS; ^$$$^*p*<0.001, rapamycin vs. 2-DG+bafilomycin; ^%%^*p*<0.001, rapamycin vs. 2-DG+3-methyladenine.

The autophagy was increased by 2-DG or rapamycin treatment and a subsequent decrease in insulin production (Fig. 1, Fig. 2). Immunofluorescence staining data showed that 2-DG or rapamycin increased LC3-positive granules or puncta were co-localized with the increased Lamp2, indicating that autophagosomes increase under conditions of 2-DG or rapamycin (Fig. 3). Subsequently, an increase in intracellular insulin was measured in INS-1E insulinoma cells following treatment with the autophagy inhibitors, bafilomycin A1 and/or 3-methyladenine, in the presence or absence of 2-DG (Fig. 4A and B) or rapamycin (Fig. 4C and D).

## 2 Experimental design, materials and methods

### 2.1 Cell culture

INS-1E cells, a clonal pancreatic β-cell line, were obtained from Prof. Claes B. Wollheim, and processed as described previously [4]. Cells were cultured in RPMI 1640 medium (Invitrogen, Carlsbad, CA, USA) containing 11 mM glucose supplemented with 10 mM HEPES (pH 7.3), 10% heat-inactivated fetal bovine serum (FBS; Invitrogen), 50 μM β-mercaptoethanol, 1 mM sodium pyruvate, 50 μg/mL penicillin, and 100 μg/mL streptomycin at 37 °C with 5% CO_2_ in a humidified incubator.

### 2.2 Treatment with 2-DG, rapamycin, bafilomycin A1, and 3-methyladenine

INS-1E cells were cultured in a 37 °C and 5% CO_2_ incubator in RPMI 1640 medium plus 2% heat-inactivated FBS with 2-DG (5 mM) (Sigma-Aldrich, St. Louis, MO, USA), or with rapamycin (20 to 80 nM) (Calbiochem, San Diego, MO, USA), for 24 h. INS-1E cells were also cultured in a 37 °C and 5% CO_2_ incubator in RPMI 1640 medium plus 2% heat-inactivated FBS with or without 2-DG (5 mM) or rapamycin, and with or without bafilomycin A1 (100 nM) (Santa Cruz Biotechnology, Santa Cruz, CA, USA) or 3-methyladenine (10 mM) (Santa Cruz Biotechnology) for 6 h.

### 2.3 Western blot analysis

We performed as described previously [4], [5]. Cells were collected, washed twice with ice-cold phosphate buffered saline (PBS), and then resuspended in PBS containing protease inhibitors (0.1 mM phenylmethylsulfonyl fluoride, 5 µg/mL aprotinin, 5 µg/mL pepstatin A, and 1 µg/mL chymostatin) and phosphatase inhibitors (5 mM Na_3_VO_4_ and 5 mM NaF). Whole cell lysate was prepared was prepared using buffer (150 mM NaCl, 1% NP-40, 50 mM Tris–HCl, pH 7.4, 0.1 mM phenylmethylsulfonyl fluoride, 5 µg/mL aprotinin, 5 µg/mL pepstatin A, and 1 µg/mL chymostatin), followed by centrifugation at 13,000×*g* for 20 min at 4 °C. Protein concentration was determined by BCA assay (Sigma-Aldrich). Cellular proteins (40 µg) were separated by 16.5% tricine sodium dodecyl sulfate-polyacrylamide gel electrophoresis (tricine SDS-PAGE) and then transferred to a polyvinylidene difluoride (PVDF) membrane. The membrane was incubated with antibodies (diluted as indicated in parentheses) directed against the following proteins: insulin (1:500) (Santa Cruz Biotechnology); LC3 (1:1000) (Cell Signaling Technology); Beclin 1 (1:500) (Santa Cruz Biotechnology) and actin (1:1000) (Assay Designs, Ann Arbor, MI, USA). Immunoreactive proteins were visualized by exposure to X-ray film. Protein bands were analyzed by image-scanning, and optical density was measured using Image J analysis software (version 1.37; Wayne Rasband, NIH, Bethesda, MD, USA). The data were corrected for background subtraction and normalized by including actin as an internal control.

### 2.4 Immunofluorescence staining

We performed as described previously [5]. INS-1E cells grown on culture slides (BD Falcon Labware, REF 354108) were permeabilized and fixed in methanol at −20 °C for 3 min. Cells were washed with phosphate-buffered saline (PBS), blocked with 10% bovine serum albumin (Sigma-Aldrich) in PBS for 10 min, and incubated with primary antibody in blocking buffer for 1 h at room temperature (RT). Cells were hybridized with secondary antibodies for 1 h at RT. The coverslips were mounted on glass slides using Vectashield mounting medium (Vector Labs Inc., Burlingame, CA, USA). Cells were viewed under a Leica TCS SP5 confocal microscope (Leica, Microsystems CMS GmbH, Wetzlar, Germany). The following primary antibodies were used: Lamp2 (Cell Signaling Technology) and LC3 (Cell Signaling Technology). The following secondary antibodies were used: Alexa 594 (red)-conjugated anti-rabbit IgG (Vector Laboratories Inc.) and fluorescein isothiocyanate (green)-labeled anti-mouse IgG (Jackson ImmunoResearch Laboratories, West Grove, PA, USA). Cells stained with 4,6-diamidino-2-phenylindole (DAPI, Santa Cruz Biotechnology) for 10 min.

### 2.5 Statistical analysis

Significant differences were determined by ANOVA, followed by Tukey׳s test for multiple comparisons. Analysis was performed using the Prism Graph Pad v4.0 (Graph Pad Software Inc., San Diego, CA, USA). Values are expressed as means±SD of at least three separate experiments, of which a representative experiment is depicted in the figures. *P* values<0.05 were considered statistically significant.

## References

[1] Marsh B.J., Soden C., Alarcón C., Wicksteed B.L., Yaekura K., Costin A.J., Morgan G.P., Rhodes C.J. (2007). Regulated autophagy controls hormone content in secretory-deficient pancreatic endocrine beta-cells. Mol. Endocrinol..

[2] Aghaee F., Pirayesh Islamian J., Baradaran B. (2012). Enhanced radiosensitivity and chemosensitivity of breast cancer cells by 2-deoxy-d-glucose in combination therapy. J. Breast Cancer.

[3] Tanemura M., Ohmura Y., Deguchi T., Machida T., Tsukamoto R., Wada H., Kobayashi S., Marubashi S. (2012). Rapamycin causes upregulation of autophagy and impairs islets function both in vitro and in vivo. Am. J. Transplant..

[4] Yoo Y.M. (2013). Melatonin-mediated insulin synthesis during endoplasmic reticulum stress involves HuD expression in rat insulinoma INS-1E cells. J. Pineal Res..

[5] Kim H.S., Choi S.I., Jeung E.B., Yoo Y.M. (2014). Cyclosporine A induces apoptotic and autophagic cell death in rat pituitary GH3 cells. PLoS One.

