# Supplementary material for: Data of intracellular insulin protein reduced by autophagy in INS-1E cells
Source: Data Brief. 2016 Jul 14;8:1151–6. doi: 10.1016/j.dib.2016.07.008 (PMC4976643; doi:10.1016/j.dib.2016.07.008)
Supplement: Supplementary file 1 — Supplementary material [file mmc1.docx]

**Conflict of Interests**

The authors declare that there is no conflict of interests regarding the publication of this paper.

**Acknowledgement**

This research was supported by the Leading Foreign Research Institute Recruitment Program through the National Research Foundation of Korea (NRF) funded by the Ministry of Science, ICT & Future Planning (2010-00757).
